# Palmitoylethanolamide for Nickel Allergy: Plausible, Untested, and Worth Considering

**DOI:** 10.3390/biomedicines14010177

**Published:** 2026-01-14

**Authors:** Irene Palenca, Silvia Basili Franzin, Giovanni Sarnelli, Giuseppe Esposito

**Affiliations:** 1 Neuropharmacology and Behavioral Science Laboratory, Santa Lucia Foundation (IRCCS Fondazione Santa Lucia), 00185 Rome, Italy; 2 Vice-President, Italian Association of Nickel-Allergic Patients (ASIPAN), 00185 Rome, Italy; 3 Department of Physiology and Pharmacology “V. Erspamer”, Sapienza University of Rome, Piazzale Aldo Moro 5, 00185 Rome, Italy; silvia.basilifranzin@uniroma1.it (S.B.F.); giuseppe.esposito@uniroma1.it (G.E.); 4 Department of Clinical Medicine and Surgery, University of Naples “Federico II”, 80131 Naples, Italy; sarnelli@unina.it

**Keywords:** Nichel allergy, SNAS, ACD, PEA

## Abstract

Nickel allergy remains the most prevalent cause of allergic contact dermatitis worldwide, imposing a substantial socio-epidemiological and economic burden. Beyond its classical cutaneous presentation, systemic nickel allergy syndrome highlights the systemic dimension of Nickel hypersensitivity, wherein dietary nickel intake may provoke both gastrointestinal and cutaneous symptoms through mechanisms involving gut barrier impairment and mucosal immune priming. Recent evidence highlights the contribution of angiogenesis and lymph-angiogenesis to Nickel-induced allergic contact dermatitis, through crosstalk among keratinocytes, mast cells, endothelial cells, and pro-angiogenic mediators such as vascular endothelial growth factor. Against this background, we propose to revisit palmitoylethanolamide, an endogenous ALIAmide with well-documented anti-inflammatory, anti-angiogenic, and anti-allergic properties. Already studied in pain and inflammatory disorders and employed in veterinary dermatology, palmitoylethanolamide down-modulates mast cell degranulation, suppresses VEGF expression via PPAR-α/Akt/mTOR signaling, and enhances intestinal barrier integrity, acting as a promising “gatekeeper” molecule that reduces gut hyperpermeability characterizing systemic nickel allergy as well as other gut disorders with systemic consequences. This paper is presented as a viewpoint intended to highlight the untapped therapeutic potential of palmitoylethanolamide, suitable for both oral and topical administration, as a candidate to address the multifactorial pathophysiology of Nickel allergic contact dermatitis and systemic nickel allergy. Our purpose is not to provide definitive answers, but to stimulate scientific debate on its rational use within emerging gut–skin therapeutic strategies. We thus encourage future experimental and clinical studies to explore its potential integration within emerging gut–skin therapeutic paradigms.

## 1. Basic Aspects of Nickel Allergy: Etiopathogenesis, Clinical Features and Current Management

Nickel (Ni) allergy remains the most common cause of allergic contact dermatitis (ACD), with prevalence rates approaching 20% in the general population [1]. Its socio-epidemiological burden is remarkable: constant exposure through jewelry, clothing fasteners, coins, cosmetics, electronic devices, and even occupational tools leads to recurrent dermatitis, reduced quality of life [2], and significant healthcare expenditures [3]. The chronicity of the condition results in absenteeism, professional impairment, and social stigma, making Ni-allergy a true public health issue rather than a mere dermatological nuisance. Beyond its classical cutaneous presentation, systemic nickel allergy syndrome (SNAS) exemplifies the systemic nature of Ni hypersensitivity, as dietary Ni ingestion can elicit gastrointestinal symptoms, such as nausea, abdominal pain, and diarrhea, alongside cutaneous manifestations like urticaria and eczema [4,5].

Management remains largely focused on restrictive low-Ni diets, which may offer symptomatic relief [6] but are often difficult to follow, nutritionally unbalanced, and socially limiting. This unmet therapeutic need emphasizes the importance of novel interventions able to act on the immunological and angiogenic mechanisms underpinning Ni hypersensitivity, improving both patient outcomes and quality of life. Mechanistically, the systemic features of SNAS have been linked to increased gastrointestinal permeability (“leaky gut”), mucosal mast cell activation, and immune sensitization occurring at the gut barrier level [7], contributing to low-grade systemic inflammation and the amplification of both GI and cutaneous symptoms in sensitized individuals.

## 2. Pathophysiological Mechanisms of Nickel Allergy: Angiogenesis, Mast Cells and the Gut–Skin Axis

It should be emphasized that Ni allergy is characterized not only by classical immune activation but also by marked cutaneous angiogenesis, which underlies and amplifies chronic inflammatory dermatoses. At the cutaneous level, Ni directly activates innate immunity by binding to human toll like receptor—4 (TLR4)/myeloid differentiation factor—2 (MD-2), initiating nuclear factor kappa-light-chain-enhancer of activated B cells (NF-κB)–dependent transcription of inflammatory cytokines even before antigen-specific T cell activation [8]. Keratinocytes exposed to Ni upregulate pro-angiogenic factors, such as vascular endothelial growth factor (VEGF), VEGF-C, and VEGF-D, produced by keratinocytes, fibroblasts, and resident immune cells [8,9]. These factors, in turn, stimulate endothelial proliferation, vascular permeability, and lymphangiogenesis [9,10]. Consistently, elevated VEGF levels have been documented in serum and lesional skin of ACD patients, correlating with clinical severity [11]. Moreover, mast cells, abundant in allergic infiltrates, amplify this process by releasing histamine, tryptase, and VEGF, sustaining both inflammation and vascular remodeling [12]. In parallel, Ni ingestion in SNAS promotes mucosal immune priming in the GI tract, where mast cell infiltration and barrier dysfunction trigger low-grade inflammation, epithelial permeability, and systemic sensitization [13]. The so-called “gut–skin axis” thus represents a key pathogenic bridge: local angiogenesis in cutaneous lesions mirrors mucosal vascular responses in the gut, both perpetuating the allergic phenotype and explaining why Ni allergy manifests as a combined dermatological and gastrointestinal disorder [4,7]. The “leaky gut” allows greater antigen translocation and promoting systemic sensitization. Within this inflammatory environment, pro-angiogenic mediators such as VEGF are upregulated, supporting microvascular expansion and increased vascular permeability in the intestinal wall. These vascular changes amplify immune cell recruitment and sustain chronic mucosal inflammation, helping explain the frequent overlap with irritable bowel syndrome (IBS) -like GI symptoms [5]. Clinically, a low-Ni diet has been shown to reduce these symptoms, and emerging evidence indicates that combining dietary restriction with probiotics may further modulate the gut microbiota and dampen inflammatory and angiogenic signaling [4]. Recent studies identifying structural and functional defects in the duodenal mucosal barrier reinforce the concept that Ni-driven immune activation, barrier impairment, and angiogenesis are interconnected processes [5,14]. Overall, the GI angiogenic response plays a central role in maintaining inflammation and facilitating the gut–skin axis that characterizes SNAS. Nickel-TLR4 signaling initiates inflammation-driven angiogenesis, while VEGF-dependent lymphatic remodeling facilitates antigen presentation and cellular trafficking between skin, lymph nodes, and gut. This bidirectional skin–gut axis explains why Ni allergy often presents with both dermatological and GI symptoms and suggests that targeting angiogenic pathways, alongside dietary and microbiome-based approaches, may provide therapeutic benefit. Molecules acting both at the gut and dermatological side could disrupt this pathogenic communication between the gut–skin axis and pave the way for new approaches in the treatment of SNAS. Although the gut–skin axis in nickel allergy has been clinically supported by dietary challenge studies in SNAS patients showing parallel gastrointestinal and cutaneous symptom modulation, mechanistic causality remains only partially defined [4,5,12,14]. Human evidence derives mainly from low-nickel diet intervention studies and clinical observational cohorts, while experimental data link nickel exposure to mast cell activation, barrier dysfunction, and angiogenic signaling. However, fully integrated animal models combining dietary nickel challenge with quantitative gut permeability endpoints are still limited.

## 3. Palmitoylethanolamide (PEA) in Current Clinical Practice: Mechanisms and Evidence Across Indications

Autacoid Local Injury Antagonists-amides, abbreviated with the acronym ALIAmides, are endogenous bioactive lipids of the N-acylethanolamine family, synthesized “on demand” in response to injury, inflammation, or cellular stress to maintain local homeostasis and prevent excessive immune activation [15]. They are produced from membrane phospholipids and converted into free NAEs such as palmitoylethanolamide (PEA) and oleoylethanolamide (OEA), while their degradation by specific enzymes (FAAH and NAAA) tightly regulates their levels and duration of action in tissues [16,17]. Among ALIAmides, PEA is the best characterized by its anti-inflammatory, analgesic, and mast-cell-modulating properties [16,17,18]. Notably, the mast cell-modulating properties of PEA are further supported by experimental evidence in ACD, where PEA levels increase locally at sites of skin inflammation and exogenous administration significantly reduces edema, inflammatory cell infiltration, and cytokine release, as demonstrated by Petrosino et al. in a well-established murine model [19]. PEA acts through Peroxisome Proliferator-Activated Receptor-alpha (PPAR-α) activation, suppressing NF-κB-driven transcription of pro-inflammatory genes, reducing VEGF/VEGFR2 signaling, and inhibiting Akt/mTOR phosphorylation, thereby exerting anti-angiogenic effects [16,20]. In addition, it stabilizes epithelial tight-junctions, counteracts oxidative stress, and restores intestinal barrier integrity, which is crucial in conditions characterized by leaky gut [13]. Clinically, PEA has shown benefits in neuropathic pain, fibromyalgia, irritable bowel syndrome, eczema, and atopic dermatitis, with an excellent safety profile supported by multiple human clinical trials and veterinary dermatology studies [21,22,23,24,25].

PEA is the most well-known ALIAmide with a well-documented high safety profile [26]. Because it is produced physiologically PEA acts as a pro-homeostatic modulator rather than as a conventional anti-inflammatory drug, and does not induce immunosuppression. Clinical and preclinical data show very low toxicity, good tolerability, and no evidence of dependency or withdrawal [25]. From a pharmacokinetic perspective, PEA is mainly degraded by NAAA and FAAH, and clinically relevant CYP-mediated drug–drug or drug-nutrients interactions have not been convincingly documented [16,25,26]. At the molecular level, PEA constitutes a heterogeneous family of endogenous lipid mediators with specific biochemical targets and therapeutic potential (Table 1). Along this line, PEA exemplifies a broad spectrum, immune regulation, mast cell modulation, barrier protection, angiogenesis inhibition, metabolic control, and satiety signaling, making it an attractive tool for chronic inflammatory and metabolic conditions, including Ni allergy, where mast cell activation, vascular remodeling, and gut–skin crosstalk converge.

### Current Clinical Use of PEA: Topical and Systemic Indications

PEA is currently used in clinical practice in both topical and oral formulations across a range of inflammatory and pain-related conditions, supported by an overall favorable safety profile [25,26]. Topical PEA preparations, including creams and emollients, have been mainly evaluated in dermatological conditions characterized by inflammation, pruritus, and barrier dysfunction, such as eczema and atopic dermatitis, where they exert predominantly local effects with good tolerability [21,22,24,29]. Oral PEA, typically administered in micronized or ultramicronized formulations, has been investigated primarily in chronic and neuropathic pain conditions, with additional evidence supporting effects on gastrointestinal barrier function and inflammation-related endpoints [13,23,25]. Table 2 summarizes the current clinical indications for PEA, distinguishing topical and systemic formulations based on available evidence.

## 4. Rationale for the Use of PEA in Ni Allergy: Skin and Gut Target Engagement

**Figure 1:**
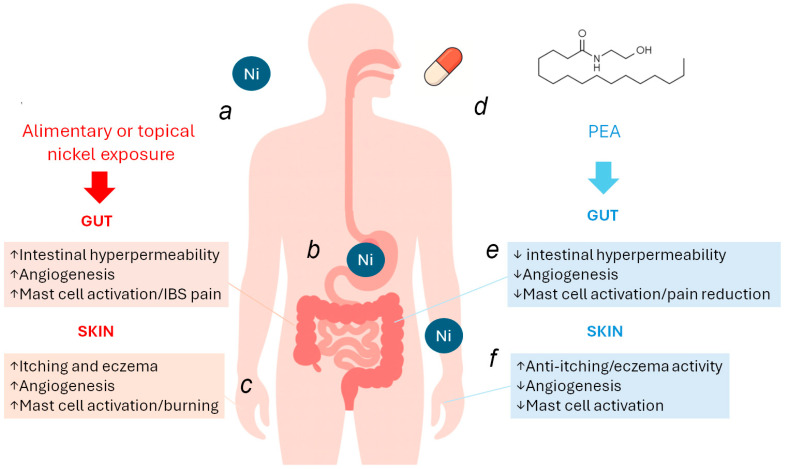
Potential dual protective effect of PEA on gastrointestinal and cutaneous manifestations caused by nickel allergy. Exposure to nickel from foods or from skin contact (a) induces a state of intestinal hyperpermeability, accompanied by angiogenesis and intestinal mast-cell sensitization, which resembles and contributes to IBS-like symptoms (b). At the cutaneous level, nickel-induced inflammation and angiogenesis are likewise associated with mast-cell activation, leading to itching, eczema, and burning sensations (c). PEA, both orally and topically (d), can markedly alleviate these symptoms. In the gut, acting as a gate-keeper molecule, it reduces intestinal hyperpermeability and provides strong control over angiogenesis and mast-cell sensitization, exerting analgesic effects and reducing abdominal discomfort (e). These actions extend to the skin as well, both with oral and topical administration, resulting in reduced cutaneous inflammation, burning, and nickel-induced rash, while also limiting angiogenesis and mast-cell release (f).

Since PEA is an endogenous fatty acid amide that regulates inflammatory and pain signaling primarily through PPAR-α activation and mast cell down-modulation [25], its application in dermatology is particularly relevant. The skin expresses components of the endocannabinoid-related system, including PEA, which contributes to epidermal barrier homeostasis and local immune control [16]. Clinically, a randomized, double-blind, vehicle-controlled trial demonstrated that a PEA-containing emollient significantly reduced pruritus and improved clinical severity in eczema with excellent tolerability [33]. In parallel, PEA has also been shown to strengthen epithelial tight-junctions and reduce mast cell-driven mucosal inflammation in GI models relevant to SNAS [27,37], supporting a gut–skin axis mechanism in SNAS and Ni-related dermatoses (Figure 1). Current clinical investigation further reflects this therapeutic interest: NCT05003453 is evaluating a 1.5% topical PEA cream for atopic dermatitis; NCT05877170 is assessing PEA in inflammatory skin conditions [24,38]. While early data consistently indicate benefits in barrier repair, pruritus control, and neuro-immune modulation, larger, longer-duration dermatology-focused trials are needed to fully define optimal formulations and treatment algorithms. Consistent with these observations, PEA’s ability to stabilize both the skin barrier and the intestinal barrier positions it as a dual-acting mediator within the gut–skin axis, capable of addressing the dermatological manifestations of ACD and the mucosal immune dysregulation driving systemic Ni hypersensitivity. Thus, both topical and oral PEA can be conceptually integrated into therapeutic strategies aimed at protecting barrier function, modulating mast cell activity, and mitigating the cutaneous and GI consequences of Ni allergy.

## 5. Advantages, Limitations and Future Directions for PEA in Ni Allergy

Current standard-of-care for Ni allergy, including ACD and, in selected cases, SNAS, relies primarily on strict Ni avoidance, together with topical corticosteroids for acute flares and topical calcineurin inhibitors as steroid-sparing options. Severe or refractory cases may require short courses of systemic corticosteroids or other immunosuppressive approaches, while low-Ni diet and oral Ni hyposensitization/immunotherapy have been explored for systemic forms of the disease [1,4]. However, these strategies present important limitations: avoidance is often impractical and does not modify the underlying disease mechanisms; corticosteroids are effective in the short term but unsuitable for long-term use due to local adverse effects and, when systemic, broader toxicities; and oral Ni immunotherapy shows variable efficacy and tolerability and remains applicable only to selected patients [1,4,6].

Unlike conventional long-term therapies, particularly corticosteroids or systemic immunosuppressants, which are often limited by cumulative toxicity and tolerability issues, PEA may allow sustained symptom control with fewer safety constraints, supporting its suitability as a candidate adjunctive strategy for chronic allergic conditions such as Ni allergy, pending dedicated clinical validation [16,24,25,26].

**Figure 2:**
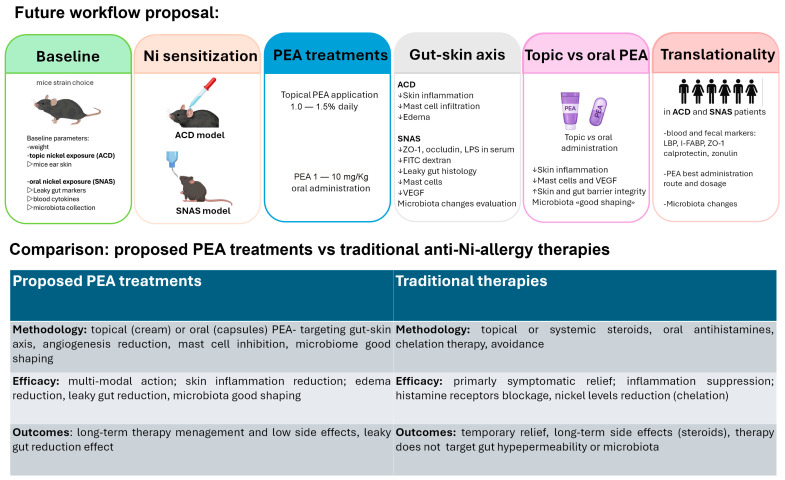
Proposed mechanistic and translational workflow for evaluating PEA in nickel allergy and SNAS. Legend: ↓ denotes a decrease, whereas ↑ denotes an increase.

Among ALIAmides, PEA was prioritized in this viewpoint because it is the most extensively characterized compound in this class, with direct preclinical evidence in ACD, established clinical use in inflammatory conditions, and commercially available oral and topical formulations, making it the most mature candidate for Ni allergy and SNAS. The potential use of PEA in Ni allergy rests on a strong mechanistic rationale but faces important translational challenges. On the positive side, PEA has an excellent safety profile, supported by decades of use in humans and veterinary medicine, and is generally well tolerated [21,22,23,25]. Its multimodal action, mast cell modulation [16,18], angiogenesis inhibition via PPAR-α/mTOR signaling [39], and gut barrier protection [13], aligns closely with the central mechanisms of both ACD and SNAS. Although anti-angiogenic strategies may raise concerns in specific clinical contexts such as pregnancy or active wound healing, PEA should not be considered a classical anti-angiogenic agent but rather a context-dependent modulator of inflammation-associated angiogenic signaling. Nevertheless, future studies should explicitly consider these conditions as exclusion criteria and include basic safety monitoring, such as wound healing assessment and circulating VEGF levels, when evaluating PEA in SNAS. The availability of multiple formulations represents another advantage: oral micronized and ultra-micronized preparations improve absorption [23], topical creams and gels are effective in eczema and atopic dermatitis [24], and novel carriers such as nano-formulations are under active investigation. Moreover, its dual intestinal and cutaneous efficacy positions PEA as an ideal candidate for targeting the gut–skin axis in systemic Ni allergy (Table 1). Importantly, PEA is not proposed as a direct microbiome- or strain-specific modulator. Rather, its potential relevance to microbiome-related processes in SNAS is framed in terms of host-directed mechanisms, including intestinal barrier restoration and immune modulation, which may secondarily influence microbiome-associated functional outputs. The proposed combined topical and oral use of PEA is based on compartment-specific mechanisms along the gut–skin axis, whereby topical formulations primarily exert local cutaneous effects, while oral micronized formulations provide intestinal and systemic availability sufficient for mucosal immune and barrier modulation. On the other hand, major limitations must be acknowledged. To date, no clinical trial has directly evaluated PEA in Ni-induced ACD or SNAS, meaning its efficacy remains hypothetical. The heterogeneity of Ni allergy phenotypes, ranging from localized dermatitis to systemic syndromes, complicates trial design and patient stratification [4,5,6]. Oral bioavailability, though improved, remains variable, and inter-individual differences in metabolism may limit efficacy [23]. From a regulatory perspective, PEA is classified mainly as a nutraceutical or cosmetic; thus, therapeutic claims would require robust randomized controlled trials, which are costly and time-consuming. Finally, the proposed additive role of PEA requires careful evaluation, since current standard management, primarily low-Ni diets, already provides clinical benefit, and it remains to be demonstrated whether PEA can offer meaningful incremental efficacy. Within this context of phenotypic heterogeneity and translational uncertainty, a mechanistically informed stratification framework becomes essential for any future evaluation of PEA in SNAS. To enable mechanistic validation and translational consistency, future studies evaluating PEA in SNAS should integrate functional measures of intestinal permeability with circulating and tissue-level biomarkers of epithelial integrity and immune activation. Table 3 summarizes candidate biomarkers and assays applicable across preclinical and clinical settings. A proposed mechanistic research workflow integrating preclinical and clinical validation steps is summarized in Figure 2.

## 6. Conclusions

Ni allergy and SNAS represent complex inflammatory conditions in which immune activation, mast cell signaling, angiogenesis, and intestinal barrier dysfunction converge along a gut–skin axis. Despite their high prevalence, current management strategies remain largely based on avoidance measures and dietary restriction, underscoring the need for adjunctive, mechanism-based approaches. Within this framework, PEA emerges as a biologically plausible and translationally accessible candidate. Its established safety profile, combined with documented anti-inflammatory, mast cell–modulating, anti-angiogenic, and barrier-protective properties, supports its consideration as an adjunct strategy for both cutaneous and gastrointestinal manifestations of Ni hypersensitivity. At the same time, the absence of direct clinical trials in Ni allergy or SNAS represents a critical gap. Future research should prioritize well-designed preclinical models and randomized controlled trials specifically addressing Ni-induced disease, incorporating objective biomarkers of barrier integrity and endotoxin-driven inflammation, together with standardized dermatological and gastrointestinal outcomes. Such studies will be essential to determine whether the mechanistic promise of palmitoylethanolamide translates into meaningful clinical benefit for patients with Ni allergy.

## Figures and Tables

**Table 1:** Therapeutic rationale and limitations of PEA in Ni allergy and SNAS. A single dash (—) indicates that the item is not applicable or that no specific experimental or clinical evidence is currently available.

Aspect	Key Points	Mechanistic/Clinical Implications	Type of Evidence/Model	References
Safety and tolerability	PEA shows an excellent safety profile in humans and veterinary applications.	Supports use in chronic and relapsing conditions without significant adverse events.	Clinical in vivo (veterinary), Preclinical in vivo	[21,22,23]
Mast cell modulation	PEA down-regulates mast cell degranulation and related inflammatory mediators.	Relevant for both cutaneous ACD lesions and gastrointestinal symptoms in SNAS.	Preclinical in vivo, Preclinical in vitro	[16,18]
Anti-angiogenic activity	PEA inhibits VEGF-driven angiogenesis via PPAR-α/Akt/mTOR signaling pathways.	Targets vascular remodeling associated with chronic ACD inflammation.	Human translational study, ex vivo mast cell activation, in vivo acid challenge	[27]
Gut barrier protection	PEA reduces intestinal permeability and restores tight junction function.	Supports the treatment of SNAS, where “leaky gut” and mucosal sensitization are central.	Human translational study, in vitro inflammatory epithelial models, ex vivo human intestinal mucosa	[13]
Formulation versatility	Available as oral micronized and ultra-micronized forms; effective topical preparations also exist.	Allows combined oral + topical treatment targeting both intestinal and skin inflammation.	Preclinical in vivo	[23,24]
Innovative delivery systems	Nano-formulations and enhanced bioavailability systems are emerging.	Potential to improve absorption and systemic distribution in SNAS patients.	Preclinical in vivo	[28]
Alignment with gut–skin axis model	PEA simultaneously modulates immune activation in skin and intestine.	Rational candidate for integrated therapeutic strategies in systemic nickel allergy.	—	Conceptual alignment
Lack of direct clinical trials	No published RCTs or preclinical models specifically testing PEA in Ni hypersensitivity.	Efficacy remains theoretical; further investigation is required.	—	—
Clinical phenotype variability	Ni allergy ranges from localized dermatitis to systemic disease.	Trial design and patient stratification are complex.	Human clinical interventional and observational studies	[4,6]
Variable oral bioavailability	Although improved by micronization, absorption remains patient-dependent.	May limit predictable systemic therapeutic effects.	Preclinical in vivo	[23]
Regulatory limitations	PEA is primarily classified as a nutraceutical/cosmetic.	Therapeutic claims require rigorous clinical validation.	—	—
Unclear additive efficacy	Low-nickel diet and avoidance often provide clinical benefit.	Need to determine whether PEA provides incremental improvement.	—	—

**Table 2:** Overview of main clinical applications of PEA in humans, according to route of administration and type of evidence.

Clinical Application	Route of Administration	Type of Human Evidence	Main Observed Effects	Representative PubMed References
Atopic dermatitis/inflammatory skin conditions	Topical	Randomized controlled clinical trial	Reduction in erythema, pruritus, skin dryness; improvement of skin barrier function compared with vehicle/emollients	[24]
Asteatotic eczema/xerosis with pruritus	Topical	Randomized, double-blind clinical trial	PEA improved hydration, reduced itching and inflammation	[30]
Chronic inflammatory dermatoses	Topical	Clinical observational and interventional study	Anti-inflammatory and antipruritic effects; good tolerability	[31]
Localized musculoskeletal or joint pain	Oral	Randomized, double-blind controlled study	Reduction in local pain intensity without relevant adverse effects	[29,32]
Chronic pain	Oral	Randomized controlled trials, meta-analyses	Significant reduction in pain scores; improvement in functional status and quality of life	[33]
Neuropathic pain	Oral	case series	Decrease in neuropathic pain intensity, often as add-on therapy	[34]
Low back pain/sciatic pain	Oral	Controlled clinical studies	Reduction in pain and disability scores compared with baseline or standard care	[35]
Adjunct treatment in multiple sclerosis	Oral	Clinical study	Reduction in injection-related pain and discomfort	[36]

**Table 3:** Candidate biomarkers and functional assays for mechanistic and translational evaluation of PEA effects on the gut–skin axis in SNAS.

Axis Level	Biomarker/Test	Pathophysiological Meaning	Preclinical (Ni-ACD/SNAS)	Clinical (Nickel Allergy)	Relevance for PEA
Gut permeability	FITC–dextran	Intestinal barrier disruption	●●●	–	Reference for PEA efficacy in restoring barrier [13]
	Lactulose/Mannitol ratio	Functional intestinal leak	●●	●●●	Non-invasive readout of systemic PEA effects [13]
Epithelial integrity	ZO-1/Occludin	Tight-junction preservation	●●●	–	Mechanistic target of PEA [40]
	I-FABP	Enterocyte damage	●●	●●	Early indicator of gut protection [41]
Microbial translocation	LPS	Bacterial product leakage	●●	●●	Functional marker of barrier failure [42,43]
	LBP	Chronic endotoxin exposure	●●	●●●	Stable translational endpoint
	sCD14	Innate immune activation	●●	●●●	Links leaky gut to skin inflammation
Immune–mast cell axis	Mast cell tryptase/histamine	Barrier and vascular modulation	●●●	●●	Direct PEA target (mast cell control) [12,27]
Angiogenic signaling	VEGF (serum/tissue)	Vascular permeability, edema	●●	●●	Shared gut–skin inflammatory mediator [9,10,11,33]
Inflammation	Fecal calprotectin	Intestinal inflammation	●●	●●●	Stratification of SNAS severity [5]
Microbiota function	SCFAs	Barrier-supportive metabolism	●●	●●	Indirect systemic PEA effect [4]

Legend: ●●● = high applicability, ●● = moderate applicability, – = not applicable.

## Data Availability

No new data were created or analyzed in this study.

## Abbreviations

The following abbreviations are used in this manuscript:

ACD	Allergic Contact Dermatitis
ALIAmides	Autacoid Local Injury Antagonist amides
FAAH	Fatty Acid Amide Hydrolase
GI	Gastrointestinal
IBD	Inflammatory Bowel Disease
IBS	Irritable Bowel Syndrome
MD-2	Myeloid Differentiation factor 2
mTOR	Mechanistic Target of Rapamycin
NAE	N-acylethanolamine
NAAA	N-acylethanolamine-hydrolyzing acid amidase
NF-κB	Nuclear Factor kappa-light-chain-enhancer of activated B cells
Ni	Nickel
OEA	Oleoylethanolamide
PEA	Palmitoylethanolamide
PPAR-α	Peroxisome Proliferator-Activated Receptor-alpha
RCT	Randomized Controlled Trial
SNAS	Systemic Nickel Allergy Syndrome
TLR4	Toll-Like Receptor 4
VEGF	Vascular Endothelial Growth Factor
